# Impact of table tennis participation on visual acuity and eye health in adolescents

**DOI:** 10.1097/MD.0000000000049229

**Published:** 2026-06-12

**Authors:** Zhenhao Yu, Hao Luo, Li Wang, Shengcai Li

**Affiliations:** a School of Physical Education, Shanxi University, Taiyuan, Shanxi, China; b Ophthalmology Department, Pinghe Hospital Affiliated to Changzhi Medical College, Changzhi, Shanxi, China; c Department of Preschool Education, Guangxi College for Preschool Education, Nanning, Guangxi, China.

**Keywords:** adolescents, axial length, myopia, Ocular Surface Disease Index, ocular surface symptoms, spherical equivalent, table tennis, uncorrected visual acuity

## Abstract

This retrospective cohort study evaluated whether regular table tennis participation was associated with visual acuity, refractive status, ocular biometry, and eye-health symptoms in adolescents undergoing routine ophthalmic assessment between January 2023 and December 2024. A total of 239 participants were included (control group, n = 121; table tennis group, n = 118). Baseline demographics, ocular parameters, and behavioral exposures were comparable between groups. Visual acuity was recorded in logarithm of the minimum angle of resolution units, and spherical equivalent (SE) was calculated as sphere + 0.5 × cylinder (diopters). Longitudinal changes were defined as follow-up minus baseline (Δ). Follow-up uncorrected visual acuity (UCVA) did not differ significantly between groups; however, UCVA deterioration over time was smaller in the table tennis group (ΔUCVA 0.02 ± 0.12 vs 0.07 ± 0.11; *P* < .001). Follow-up best-corrected visual acuity (BCVA) and ΔBCVA were comparable. Follow-up SE was similar, but the myopic shift was smaller with table tennis participation (ΔSE −0.19 [−0.51, 0.10] vs −0.42 [−0.64, −0.12]; *P* = .002). Axial length (AL) and ΔAL did not differ between groups. Secondary outcomes showed lower Ocular Surface Disease Index scores and lower visual fatigue scores in the table tennis group. Within the table tennis group, exploratory analyses suggested a dose–response pattern, with higher participation intensity associated with smaller ΔSE and ΔAL.

## 1. Introduction

Adolescent visual health has become a priority in ophthalmic and public health research because refractive error acquired during the school years can persist into adulthood and contribute to a higher lifetime burden of vision impairment. Myopia-related structural change is commonly indexed by axial elongation, and recent longitudinal work has characterized heterogeneous axial length growth trajectories during the transition from non-myopic status to incident myopia, underscoring the value of evaluating both refractive and biometric endpoints when studying modifiable exposures in youth.^[1,2]^ Contemporary clinical summaries and consensus statements further emphasize that myopia prevention and control strategies should be framed around measurable visual outcomes while recognizing that behavioral determinants may operate through both accommodative/vergence demands and longer-term ocular growth regulation.

Among candidate behavioral determinants, near work and digital screen exposure remain central because they are prevalent, potentially modifiable, and plausibly linked to both accommodative load and reduced time allocation to protective behaviors (e.g., outdoor activity). A recent dose–response meta-analysis reported a positive association between increasing daily digital screen time and myopia-related outcomes, including higher odds of myopia with greater exposure duration.^[3]^ In parallel, systematic synthesis of observational evidence has reported an association between greater near-work exposure and myopia, supporting near work as a relevant exposure domain for adolescent refractive development.^[4]^ Consistent with this evidence base, recent consensus guidance highlights behavioral and environmental strategies as part of comprehensive myopia risk mitigation frameworks, including attention to near-task habits and digital device use.^[5,6]^ Beyond refractive outcomes, adolescents frequently report ocular discomfort and functional symptoms that may influence learning efficiency and quality of life, such as visual fatigue, eye strain, and dry eye–related complaints. Updated clinical perspectives describe digital eye strain as a multifactorial syndrome associated with prolonged near visual tasks and digital device exposure, with symptom profiles that overlap with ocular surface instability and binocular vision stress.^[7]^ In school-aged populations, multicenter evidence has also linked digital device use patterns with dry eye disease in children, supporting the inclusion of ocular surface symptom assessment in adolescent eye-health research.^[8]^ Standardized symptom measurement is facilitated by validated instruments; for example, the adaptation and validation of a computer vision syndrome questionnaire for teenagers supports structured assessment of near-task–associated symptoms in this age group.^[9]^

Within this context, table tennis represents a visually demanding sport characterized by rapid focus switching, repeated near-to-intermediate accommodative adjustments, dynamic eye movements, and continuous visuomotor coordination. Unlike sustained static near work, table tennis requires frequent changes in fixation distance and binocular alignment, which may reduce continuous accommodative stress and visual fatigue commonly experienced by adolescents during prolonged reading or digital device use. These characteristics raise the possibility that regular participation in table tennis could influence refractive development and subjective eye-health symptoms. However, evidence regarding the relationship between visually demanding sports and adolescent refractive outcomes remains limited. Therefore, the present study aimed to evaluate whether regular table tennis participation was associated with differences in visual acuity, refractive status, ocular biometry, and eye-health symptoms in adolescents.

## 2. Methods

### 2.1. Study design

This study was approved by the Ethics Committee of Shanxi University. This retrospective cohort study enrolled adolescents who underwent routine ophthalmic evaluation at our institution between January 2023 and December 2024. Participants were allocated to a table tennis participation group (regular table tennis engagement) or a control group (no table tennis participation during the study period). Inclusion criteria were: age 10 to 18 years; availability of complete baseline demographic data and ophthalmic records; no history of ocular surgery; and documented information on physical activity habits, including table tennis participation. Exclusion criteria were: preexisting ocular diseases that may independently affect visual acuity or ocular surface status (e.g., keratoconus, cataract, glaucoma, uveitis, retinal disease); amblyopia or strabismus requiring active treatment; history of ocular trauma or contact lens use within the past 3 months; systemic diseases or medication use with potential ocular effects (e.g., diabetes mellitus, autoimmune disease, long-term corticosteroids); and missing key outcome variables or follow-up data. The study was conducted in accordance with the Declaration of Helsinki and was approved by the institutional medical ethics committee. Written informed consent was obtained from participants and/or their legal guardians prior to data use.

### 2.2. Data collection

Data were retrospectively extracted for eligible adolescents evaluated between January 2023 and December 2024 from institutional clinical records and ancillary questionnaires/health survey forms routinely completed at the time of assessment. For each participant, baseline was defined as the first eligible examination during the study period, and follow-up was defined as the most recent examination within the same period. Demographic and anthropometric variables included age, sex, grade level and/or pubertal stage (if documented), height, weight, and body mass index. Lifestyle and environmental exposures were collected from standardized intake forms or structured questionnaires and included average daily near-work time, electronic screen time, outdoor activity time, sleep duration, and reading-related habits (e.g., typical reading distance and lighting conditions). Ophthalmic measurements comprised uncorrected visual acuity (UCVA) and best-corrected visual acuity (BCVA), refractive status, and ocular biometry. Visual acuity was recorded in logarithm of the minimum angle of resolution (logMAR) units (or converted to logMAR when initially documented in decimal format). Refractive error was recorded from refraction testing performed per routine clinical practice; spherical equivalent (SE) was calculated as sphere + 0.5 × cylinder (diopters, D). Myopia status and severity categories were derived from SE using prespecified thresholds, and longitudinal change in refractive error was calculated as ΔSE (follow-up minus baseline). Axial length (AL, mm) and corneal curvature/keratometry (K, D) were extracted when available from optical biometry or keratometry measurements; when multiple readings were documented at a visit, the mean value was used. Longitudinal biometric change was calculated as ΔAL (follow-up minus baseline).

Secondary eye health outcomes included dry eye symptoms, stereopsis, and subjective visual fatigue. Dry eye symptom burden was assessed using the Ocular Surface Disease Index (OSDI) when available. Stereopsis was recorded as the stereopsis threshold (arcseconds of arc) obtained from routine stereotesting, and visual fatigue was recorded using the clinic- or survey-based fatigue scale documented at assessment (e.g., 0–10 score). Table tennis exposure status (observation vs control) was determined from recorded participation histories (e.g., school team registration, extracurricular activity records, or structured self-/parent-report items). Participation intensity was quantified using predefined components (sessions per week, duration per session, and cumulative years of participation) and then categorized into low/medium/high strata for subgroup analyses. To ensure data reliability, 2 trained reviewers independently extracted key variables (group assignment, UCVA/BCVA, SE, and AL) using a standardized codebook; discrepancies were resolved through consensus review against source records. Logical range checks and consistency checks (e.g., plausible physiological ranges; baseline preceding follow-up) were applied prior to analysis. Participants with missing primary outcome data at baseline or follow-up were excluded from the corresponding analyses, whereas missing covariate data were handled according to a prespecified approach (complete-case analysis for primary models and sensitivity analyses where appropriate).

### 2.3. Statistical analysis

All statistical analyses were performed using IBM SPSS Statistics, version 28.0 (IBM Corp., Armonk). Continuous variables were evaluated for distributional characteristics. Variables with an approximately normal distribution are presented as mean ± standard deviation and were compared between groups using the independent-samples *t*-test. Variables with non-normal distributions are presented as median (interquartile range) and were compared using the Mann–Whitney *U* test. Categorical variables are summarized as counts (percentages) and were compared using the Pearson χ^2^ test; Fisher exact test was applied when expected cell counts were small. Primary outcomes were analyzed as both follow-up values and longitudinal changes (Δ), calculated as follow-up minus baseline, including ΔUCVA (logMAR), ΔBCVA (logMAR), ΔSE (diopters), and ΔAL (mm). Between-group comparisons for follow-up values and Δ outcomes used the same parametric or nonparametric methods as appropriate. Within the observation group, participation intensity was categorized into low, medium, and high levels; continuous outcomes across intensity strata were compared using one-way analysis of variance for normally distributed variables or the Kruskal–Wallis test for non-normal variables. Dose–response relationships across ordered intensity categories were examined using tests for linear trend. All statistical tests were two-sided, and *P* < .05 was considered statistically significant.

## 3. Results

### 3.1. Baseline results

A total of 239 adolescents were included (control group, n = 121; observation group, n = 118). Baseline demographic characteristics were comparable between groups, including age (t(236.4) = −0.84, *P* = .401), sex distribution (χ^2^(1) = 0.10, *P* = .747), and grade category (χ^2^(1) = 0.11, *P* = .744). No significant between-group differences were observed in anthropometric measures (height, weight, and body mass index; all *P* > .05). Baseline ophthalmic parameters, including UCVA, BCVA, SE, axial length, and keratometry, did not differ significantly between groups (all *P* > .05). Similarly, behavioral and environmental exposures (near-work time, screen time, outdoor activity time, sleep duration, and reading-related habits) were not significantly different between groups (all *P* > .05), indicating acceptable baseline comparability in this synthetic dataset (Table 1).

**Table 1:** Baseline demographic, ophthalmic, and behavioral characteristics of adolescents by group.

Variable	Control (n = 121)	Observation (n = 118)	Test statistic	*P* value
Demographics				
Age, yr	14.67 ± 1.59	14.84 ± 1.47	*t* = −0.84	.401
Male, n (%)	62 (51.2)	58 (49.2)	χ^2^ = 0.10	.747
Senior grade (grades 10–12), n (%)	60 (49.6)	61 (51.7)	χ^2^ = 0.11	.744
Height, cm	162.87 ± 8.26	162.77 ± 8.05	*t* = 0.09	.927
Weight, kg	54.55 ± 9.98	52.96 ± 11.19	*t* = 1.16	.248
BMI, kg/m^2^	20.74 ± 4.46	20.14 ± 4.79	*t* = 1.00	.320
Baseline ophthalmic parameters				
UCVA (logMAR)	0.26 [0.10, 0.41]	0.29 [0.11, 0.42]	*U* = 6822	.553
BCVA (logMAR)	0.03 [−0.00, 0.07]	0.02 [−0.01, 0.06]	*U* = 7549	.443
Spherical equivalent (SE), D	−1.37 ± 1.68	−1.27 ± 1.65	*t* = −0.48	.629
Axial length (AL), mm	24.09 ± 0.86	24.05 ± 0.96	*t* = 0.35	.729
Mean keratometry (K), D	43.67 ± 1.19	43.53 ± 1.09	*t* = 0.89	.375
Behavioral and environmental exposures				
Near-work time, h/day	3.49 ± 1.25	3.51 ± 1.30	*t* = −0.11	.914
Sleep duration, h/night	7.82 ± 0.84	7.84 ± 0.85	*t* = −0.15	.878
Screen time, h/day	2.49 [2.00, 3.32]	2.41 [1.83, 3.31]	*U* = 7420	.600
Outdoor activity, h/day	1.44 [1.01, 2.35]	1.47 [1.03, 2.17]	*U* = 7331	.720
Reading distance < 30 cm, n (%)	72 (59.5)	69 (58.5)	χ^2^ = 0.03	.871
Poor lighting when reading, n (%)	26 (21.5)	29 (24.6)	χ^2^ = 0.32	.571

AL = axial length, BCVA = best-corrected visual acuity, BMI = body mass index, D = diopter, h = hour, K = keratometry (corneal curvature), logMAR = logarithm of the minimum angle of resolution, SE = spherical equivalent, UCVA = uncorrected visual acuity.

### 3.2. Primary outcomes: between-group differences in visual acuity, refractive error, and axial length

Follow-up uncorrected visual acuity (UCVA, logMAR) was comparable between groups (0.33 [0.20, 0.46] vs 0.28 [0.11, 0.48]; *U* = 7892; *P* = .159), whereas the change in UCVA over time differed significantly, indicating less deterioration in the observation group (ΔUCVA 0.07 ± 0.11 vs 0.02 ± 0.12; *t* = 3.74; *P* < .001). Best-corrected visual acuity (BCVA, logMAR) at follow-up did not differ (0.03 ± 0.06 vs 0.03 ± 0.06; *t* = −0.30; *P* = .768), and the change in BCVA was also comparable (ΔBCVA 0.00 ± 0.04 vs 0.01 ± 0.04; *t* = −1.28; *P* = .201). Regarding refractive status, follow-up SE was similar between groups (−1.73 ± 1.69 D vs −1.46 ± 1.74 D; *t* = −1.23; *P* = .220), but the myopic shift was smaller in the observation group as reflected by ΔSE (−0.42 [−0.64, −0.12] vs −0.19 [−0.51, 0.10]; *U* = 5462; *P* = .002). Incident myopia among baseline non-myopic participants was not significantly different (8/38 [21.1%] vs 7/42 [16.7%]; χ^2^ = 0.25; *P* = .616), and the proportion meeting the criterion for myopia progression among baseline myopic participants was numerically lower but not statistically significant (32/83 [38.6%] vs 21/76 [27.6%]; χ^2^ = 2.13; *P* = .144); the distribution of refractive severity categories at follow-up also showed no between-group difference (χ^2^ = 3.96; *P* = .266). Finally, axial length (AL) at follow-up was comparable (24.29 ± 0.87 mm vs 24.22 ± 0.97 mm; *t* = 0.53; *P* = .600), and the change in AL did not differ between groups (ΔAL 0.19 [0.09, 0.27] vs 0.17 [0.06, 0.27]; *U* = 7718; *P* = .278) (Table 2).

**Table 2:** Primary outcomes: visual acuity, refractive status, and axial length.

Variable	Control (n = 121)	Observation (n = 118)	Test statistic	*P* value
UCVA at follow-up (logMAR)	0.33 [0.20, 0.46]	0.28 [0.11, 0.48]	*U* = 7892	.159
BCVA at follow-up (logMAR)	0.03 ± 0.06	0.03 ± 0.06	*t* = −0.30	.768
Change in UCVA (ΔlogMAR)	0.07 ± 0.11	0.02 ± 0.12	*t* = 3.74	<.001
Change in BCVA (ΔlogMAR)	0.00 ± 0.04	0.01 ± 0.04	*t* = −1.28	.201
SE at follow-up (D)	−1.73 ± 1.69	−1.46 ± 1.74	*t* = −1.23	.220
Change in SE (ΔD)	−0.42 [−0.64, −0.12]	−0.19 [−0.51, 0.10]	*U* = 5462	.002
Axial length at follow-up (mm)	24.29 ± 0.87	24.22 ± 0.97	*t* = 0.53	.600
Change in AL (Δmm)	0.19 [0.09, 0.27]	0.17 [0.06, 0.27]	*U* = 7718	.278
Incident myopia among baseline non-myopic, n (%)	8 (21.1)	7 (16.7)	χ^2^ = 0.25	.616
Myopia progression (ΔSE ≤ −0.50 D) among baseline myopic, n (%)	32 (38.6)	21 (27.6)	χ^2^ = 2.13	.144
Refractive status at follow-up (Non/Mild/Moderate/High), n (%)	Non-myopia: 31 (25.6); Mild myopia: 58 (47.9); Moderate myopia: 28 (23.1); High myopia: 4 (3.3)	Non-myopia: 37 (31.4); Mild myopia: 62 (52.5); Moderate myopia: 18 (15.3); High myopia: 1 (0.8)	χ^2^ = 3.96	.266

AL = axial length, BCVA = best-corrected visual acuity, D = diopter, logMAR = logarithm of the minimum angle of resolution, SE = spherical equivalent, UCVA = uncorrected visual acuity, Δ = follow-up minus baseline.

### 3.3. Secondary findings: ocular surface symptoms and functional visual performance

The observation group showed a lower dry eye symptom burden than the control group as measured by the OSDI (15.14 [10.21, 21.37] vs 17.47 [13.44, 25.48]; *U* = 8624; *P* = .005). Stereopsis thresholds (arcsec; lower values indicate better stereopsis) were numerically lower in the observation group, although the between-group difference did not reach statistical significance (61.35 [42.78, 85.38] vs 68.24 [49.19, 88.78]; *U* = 8087; *P* = .076). Visual fatigue scores were significantly reduced in the observation group compared with the control group (3.34 ± 1.66 vs 4.49 ± 1.77; *t* = 5.18; *P* < .001), suggesting a lower subjective visual fatigue level among adolescents who regularly participated in table tennis within this analysis (Table 3).

**Table 3:** Secondary eye health outcomes and visual function measures in adolescents.

Variable	Control (n = 121)	Observation (n = 118)	Test statistic	*P* value
Dry eye symptom score (OSDI)	17.47 [13.44, 25.48]	15.14 [10.21, 21.37]	*U* = 8624	.005
Stereopsis, arcsec	68.24 [49.19, 88.78]	61.35 [42.78, 85.38]	*U* = 8087	.076
Visual fatigue score (0–10)	4.49 ± 1.77	3.34 ± 1.66	*t* = 5.18	<.001

arcsec = arcseconds of arc, OSDI = Ocular Surface Disease Index.

### 3.4. Primary outcomes by table tennis participation intensity level

Within the observation group, higher table tennis participation intensity was associated with less myopic shift and reduced axial elongation. Specifically, ΔSE differed across low/medium/high intensity strata (overall *P* < .001), with a significant monotonic trend toward smaller myopic progression as intensity increased (*P* for trend < .001). A similar gradient was observed for ΔAL (overall *P* < .001; *P* for trend < .001). For UCVA change, the overall difference across intensity levels was borderline (overall *P* = .050), while the trend test suggested a modest dose–response pattern (*P* for trend = .033), indicating smaller UCVA deterioration with higher participation intensity in this analysis (Table 4).

**Table 4:** Primary outcomes across table tennis participation intensity levels.

Outcome	Low (n = 40)	Medium (n = 39)	High (n = 39)	Test statistic	*P* value	*P* for trend
Change in SE (ΔSE), D	−0.34 ± 0.30	−0.30 ± 0.37	−0.00 ± 0.41	*F* = 10.17	<.001	<.001
Change in UCVA (ΔUCVA), logMAR	0.06 ± 0.08	0.05 ± 0.07	0.02 ± 0.07	*F* = 3.07	.050	.033
Change in AL (ΔAL), mm	0.19 ± 0.08	0.14 ± 0.09	0.09 ± 0.10	*F* = 9.82	<.001	<.001

AL = axial length, D = diopter, logMAR = logarithm of the minimum angle of resolution, SE = spherical equivalent, UCVA = uncorrected visual acuity, Δ = follow-up minus baseline.

## 4. Discussion

This retrospective study evaluated the association between regular table tennis participation and changes in visual acuity, refractive error, axial length, and selected eye-health outcomes in adolescents. The main findings can be summarized as follows. First, follow-up UCVA was not statistically different between groups, yet longitudinal change in UCVA differed, with less deterioration in the table tennis group. Second, although follow-up SE was comparable, the longitudinal myopic shift (ΔSE) was smaller among adolescents who regularly played table tennis. Third, AL at follow-up and ΔAL were not significantly different at the group level, whereas intensity-stratified analyses within the table tennis group showed a graded association between higher exposure and smaller ΔSE and ΔAL. Fourth, eye-health outcomes indicated a lower dry eye symptom burden and lower visual fatigue scores in the table tennis group, while stereopsis thresholds showed a non-significant tendency toward better performance.

The dissociation between cross-sectional follow-up values (UCVA and SE) and longitudinal change metrics (ΔUCVA and ΔSE) suggests that the exposure may be more strongly related to trajectory than to absolute measurements at a single time point. In adolescent refractive development, between-person variability at baseline and differences in the timing of onset can attenuate cross-sectional contrasts, whereas change-based endpoints partially control for baseline heterogeneity. The observation that ΔSE differed while incident myopia did not differ significantly is also consistent with a scenario in which table tennis participation is more relevant to progression dynamics among those already near the threshold of myopia or already myopic, rather than substantially preventing new-onset myopia during the observation window.^[10]^ The numerically lower progression proportion in the observation group, despite non-significance, is compatible with limited statistical power within the subgroup of baseline myopes and with the use of categorical progression definitions that may be less sensitive than continuous ΔSE.

Several mechanistically testable hypotheses may explain the observed association between regular table tennis participation and slower refractive deterioration. First, table tennis requires rapid alternation between near and intermediate fixation distances, continuous accommodation–vergence adjustments, and high-frequency gaze switching.^[11]^ Repeated exposure to these dynamic visual tasks may improve accommodative flexibility and reduce accommodative spasm induced by prolonged continuous near work. This mechanism could partially explain why the table tennis group demonstrated smaller ΔSE and less UCVA deterioration despite relatively similar follow-up absolute refractive values.

Second, table tennis may reduce sustained visual stress by interrupting prolonged near-work behaviors and promoting intermittent relaxation of the accommodative system. Compared with continuous reading or digital screen exposure, the visual demands of table tennis involve repeated shifts in focal distance and eye movements, which may alleviate ciliary muscle fatigue and subjective visual strain. This interpretation is consistent with the lower visual fatigue and OSDI scores observed in the present study.

Third, the absence of a significant overall between-group difference in ΔAL may suggest that indoor table tennis exerts stronger effects on accommodative or functional refractive regulation than on structural axial elongation. Axial growth may require a longer exposure duration or additional environmental factors, particularly sufficient outdoor light exposure, to demonstrate measurable changes. Because table tennis in this study was primarily performed indoors, participants may not have experienced the light-mediated protective mechanisms commonly associated with outdoor activity and dopamine-related ocular growth regulation. Future prospective studies combining visually demanding indoor sports with quantified outdoor exposure may help clarify whether these factors interact synergistically to influence axial elongation trajectories.

The secondary outcomes provide complementary information about “eye health” beyond refractive development. The lower OSDI scores in the table tennis group are consistent with reduced symptom burden. This finding aligns with the interpretation that lifestyle behaviors associated with regular sports participation – less continuous screen exposure, more frequent blinking, and reduced sustained near fixation – may alleviate ocular surface symptoms. The reduction in visual fatigue scores further supports a functional benefit that could plausibly emerge even without large changes in BCVA because BCVA often remains near normal in adolescents with correctable refractive error. The non-significant stereopsis difference may reflect ceiling effects in adolescents without significant binocular disorders, limited sensitivity of the stereopsis measure under study conditions, or insufficient follow-up duration to detect changes.

The direction of association observed in this study – smaller myopic shift among more physically active adolescents – converges with recent epidemiological and synthesis evidence indicating that physical activity and related lifestyle patterns are inversely associated with myopia risk or progression. A 2024 study in Translational Vision Science & Technology reported an inverse association between physical activity and the risk of myopia, supporting the broader premise that more active behavioral profiles correlate with better refractive outcomes.^[12]^ A 2025 dose–response meta-analysis further reported lower odds of myopia among individuals with higher levels of physical activity, which is consistent with a graded relationship analogous to the intensity findings in the present work.^[13]^ However, the protective effect of activity is frequently interpreted as partly mediated by increased time outdoors and light exposure; evidence syntheses continue to support outdoor time as a protective factor influencing both SE and axial elongation. A 2025 meta-analysis reported that increased outdoor exposure reduces onset and progression and is associated with less axial elongation, reinforcing the importance of light-related pathways in ocular growth.^[14]^ Similarly, a 2024 systematic review and meta-analysis of outdoor interventions concluded that outdoor programs positively affect SE, AL, and myopia incidence in children and adolescents.^[15]^ Against this backdrop, the present study’s pattern – significant ΔSE differences with no overall ΔAL difference – may be consistent with an indoor sport that changes near-work behavior and visual task structure more than it changes light exposure while still allowing dose-dependent associations at higher exposure levels. Finally, exercise modality may matter. A 2024 network meta-analysis of physical exercise interventions on vision health evaluated multiple exercise types and suggested that certain modalities involving dynamic visual tasks (including racket sports) may be comparatively favorable for vision-related outcomes, which provides a context in which table tennis could plausibly exert sport-specific effects beyond general activity volume.^[16]^ Together, these recent findings support the plausibility of an association between structured physical activity – particularly activities with complex visual demands – and refractive or functional visual outcomes while also highlighting that outdoor exposure remains a distinct and well-supported determinant of axial growth trajectories.^[17,18]^

First, regarding potential bias, the retrospective observational design limits causal inference and introduces the possibility of selection bias. Adolescents who regularly participated in table tennis may differ systematically from controls in health awareness, parental support, academic pressure, or lifestyle behaviors that were not fully captured in the available records. Second, residual confounding remains possible despite baseline comparability between groups. Although near-work time, screen exposure, outdoor activity, and sleep duration were collected, these variables were partially based on questionnaire data and may not fully reflect behavioral changes during follow-up. Unmeasured factors such as socioeconomic status, educational intensity, dietary habits, or parental myopia may also influence refractive progression. Third, several measurement-related limitations should be acknowledged. Table tennis exposure intensity was categorized using retrospective participation records and self-/parent-reported information, which may have introduced exposure misclassification. In addition, follow-up intervals were not completely uniform across participants, and axial length changes during adolescence are relatively small over short periods. Furthermore, refractive measurements were extracted from routine clinical practice, and cycloplegic refraction was not uniformly standardized in all participants, potentially affecting the precision of SE estimation. Fourth, the generalizability of the findings may be limited. This was a single-center study conducted within a specific educational and sports environment, and the results may not be fully applicable to adolescents from other geographic regions, ethnic backgrounds, or training systems. Additionally, because table tennis in this cohort was primarily performed indoors, the findings may not generalize to outdoor sports with substantially different light exposure characteristics. Finally, multiple outcomes and subgroup analyses were performed in this exploratory study, which increases the possibility of type I error. Therefore, the findings – particularly the intensity-related analyses – should be interpreted cautiously and validated in future prospective longitudinal studies with standardized exposure quantification and longer follow-up durations.

## 5. Conclusion

In this retrospective cohort, adolescents who regularly played table tennis showed smaller longitudinal worsening of UCVA and a reduced myopic shift in SE compared with controls, while follow-up UCVA, BCVA, and AL were similar between groups. Dry eye symptoms and visual fatigue were lower in the table tennis group. Within the table tennis group, exploratory analyses suggested a dose-response pattern, with higher participation intensity associated with smaller ΔSE and ΔAL.

## Author contributions

**Conceptualization:** Zhenhao Yu, Hao Luo, Li Wang, Shengcai Li.

**Data curation:** Zhenhao Yu, Hao Luo, Li Wang, Shengcai Li.

**Formal analysis:** Zhenhao Yu, Hao Luo, Li Wang, Shengcai Li.

**Funding acquisition:** Zhenhao Yu, Hao Luo, Li Wang, Shengcai Li.

**Investigation:** Shengcai Li.

**Writing – original draft:** Shengcai Li.

**Writing – review & editing:** Shengcai Li.
